# Validity, reliability and support for implementation of independence-scaled procedural assessment in laparoscopic surgery

**DOI:** 10.1007/s00464-015-4254-2

**Published:** 2015-09-28

**Authors:** Kelvin H. Kramp, Marc J. van Det, Nic J. G. M. Veeger, Jean-Pierre E. N. Pierie

**Affiliations:** Department of Surgery, Leeuwarden Medical Center, Leeuwarden, The Netherlands; Department of Surgery, Hospital Group Twente, Almelo, The Netherlands; Department of Epidemiology, Leeuwarden Medical Center, Leeuwarden, The Netherlands; Department of Epidemiology, University Medical Center Groningen, University of Groningen, Groningen, The Netherlands; Post Graduate School of Medicine, University of Groningen, Groningen, The Netherlands

**Keywords:** Laparoscopy, Minimal invasive surgery, Surgical education, Laparoscopic cholecystectomy, Global rating scale, GOALS, OSATS, Procedure-based assessment

## Abstract

**Background:**

There is no widely used method to evaluate procedure-specific laparoscopic skills. The first aim of this study was to develop a procedure-based assessment method. The second aim was to compare its validity, reliability and feasibility with currently available global rating scales (GRSs).

**Methods:**

An independence-scaled procedural assessment was created by linking the procedural key steps of the laparoscopic cholecystectomy to an independence scale. Subtitled and blinded videos of a novice, an intermediate and an almost competent trainee, were evaluated with GRSs (OSATS and GOALS) and the independence-scaled procedural assessment by seven surgeons, three senior trainees and six scrub nurses. Participants received a short introduction to the GRSs and independence-scaled procedural assessment before assessment. The validity was estimated with the Friedman and Wilcoxon test and the reliability with the intra-class correlation coefficient (ICC). A questionnaire was used to evaluate user opinion.

**Results:**

Independence-scaled procedural assessment and GRS scores improved significantly with surgical experience (OSATS *p* = 0.001, GOALS *p* < 0.001, independence-scaled procedural assessment *p* < 0.001). The ICCs of the OSATS, GOALS and independence-scaled procedural assessment were 0.78, 0.74 and 0.84, respectively, among surgeons. The ICCs increased when the ratings of scrub nurses were added to those of the surgeons. The independence-scaled procedural assessment was not considered more of an administrative burden than the GRSs (*p* = 0.692).

**Discussion/conclusion:**

A procedural assessment created by combining procedural key steps to an independence scale is a valid, reliable and acceptable assessment instrument in surgery. In contrast to the GRSs, the reliability of the independence-scaled procedural assessment exceeded the threshold of 0.8, indicating that it can also be used for summative assessment. It furthermore seems that scrub nurses can assess the operative competence of surgical trainees.

Traditionally, assessment of trainees is based on objective but unreliable measures of surgical skills such as blood loss, operation time and perioperative complications. As an alternative, Martin et al. [1] developed the Objective Surgical Assessment of Technical Skills (OSATS). The OSATS has been validated in a series of studies and has become the golden standard for structured feedback toward trainees [2–5]. However, in the last decennia, laparoscopic surgery has become the standard of care for an increasing list of procedures. In contrast to open surgery, the performance of laparoscopic surgery requires the ability to work with a two-dimensional view, decreased degrees of freedom, reduced tactile feedback and the fulcrum effect (inversion and scaling of movements of the parts of the instruments inside the abdomen). Therefore, Vassiliou et al. [6, 7] developed Global Operative Assessment of Laparoscopic Skills (GOALS), a non-procedure-specific assessment tool that can be used to assess procedures in minimal invasive surgery (MIS). Although GRSs, such as the OSATS and GOALS, are useful tools for formative assessment (feedback during learning in low-stakes evaluation), a systematic review conducted by Van Hove et al. [4] demonstrated a lack of high-level evidence that these and other GRSs are reliable enough for summative assessment (assessment of learning in high-stakes examinations) in the OR. Furthermore, a survey among gynecological residents and gynecologists indicated that the OSATS was not considered an objective instrument for assessment [5]. In another survey, conducted by Beard et al. [8] among clinical supervisors and trainees, the greatest number of negative responses was related to the use of OSATS for summative assessment. The insufficient reliability and the negative responses about the objectivity of the OSATS in surveys are shortcomings that have been used as arguments to prohibit the use of the GRSs as tools for summative assessment in surgical education [4, 5, 8].

Procedural assessment has been proposed as an alternative to GRSs [8]. A procedural assessment method could enable clinicians to provide procedural specific feedback and, in contrast to the GRSs, could facilitate examination in the performance of a procedure. In order to be useful for these purposes, it should comply with three requirements. First, it should be a valid measure of improvement in performance level in a procedure. Second, to facilitate summative assessment, it should be a highly reliable tool in identifying trainees who can safely perform uncomplicated procedures without supervision. Third, it should have enough support from trainees and supervising surgeons to make implementation into clinical practice feasible. To our knowledge, there is no widely used procedural assessment yet that meets all these demands. Hence, our first aim was to create a procedural assessment for a procedure that is routinely performed with minimal invasive surgery, the laparoscopic cholecystectomy (LC). The second aim was to estimate the validity, reliability and support for implementation of this assessment method. The third aim was to compare the validity, reliability and support for implementation of the procedural assessment with that of the already existing GRSs.

## Materials and methods

### Development of the independence-scaled procedural assessment

A procedural assessment for the LC was developed in two phases. The first phase has recently been published and consists of twenty-one experts from the North-East Surgical School of the Netherlands that participated in an anonymous survey about the procedural key steps of the LC [9].

In the second phase, conducted in the present study, the key procedural steps were linked to a rating scale published by Glarner et al. [10] to create an independence-scaled procedural assessment for the LC. This rating scale was chosen because it was observed that in the learning situation, supervising surgeons aimed to find a balance between creating the optimal learning experience for the trainee and guarding the patient safety and flow throughout the operation. They attempted to achieve this goal with: (1) verbal guidance and (2) takeovers. Verbal guidance, consisting of instructions and corrections, was given to optimize surgical behavior. If verbal guidance insufficiently corrected the behavior of the trainee, supervising surgeons tend to take over one or both instruments to guard the safety and flow of the procedure. The independence-based assessment model used by Glarner et al. connects to this balance between patient-first mentality and creating the optimal learning environment. It is different from a Likert-type scale in that the frequency of verbal guidance and takeovers is used to quantify the quality of surgical skills.

**Fig. 1:**
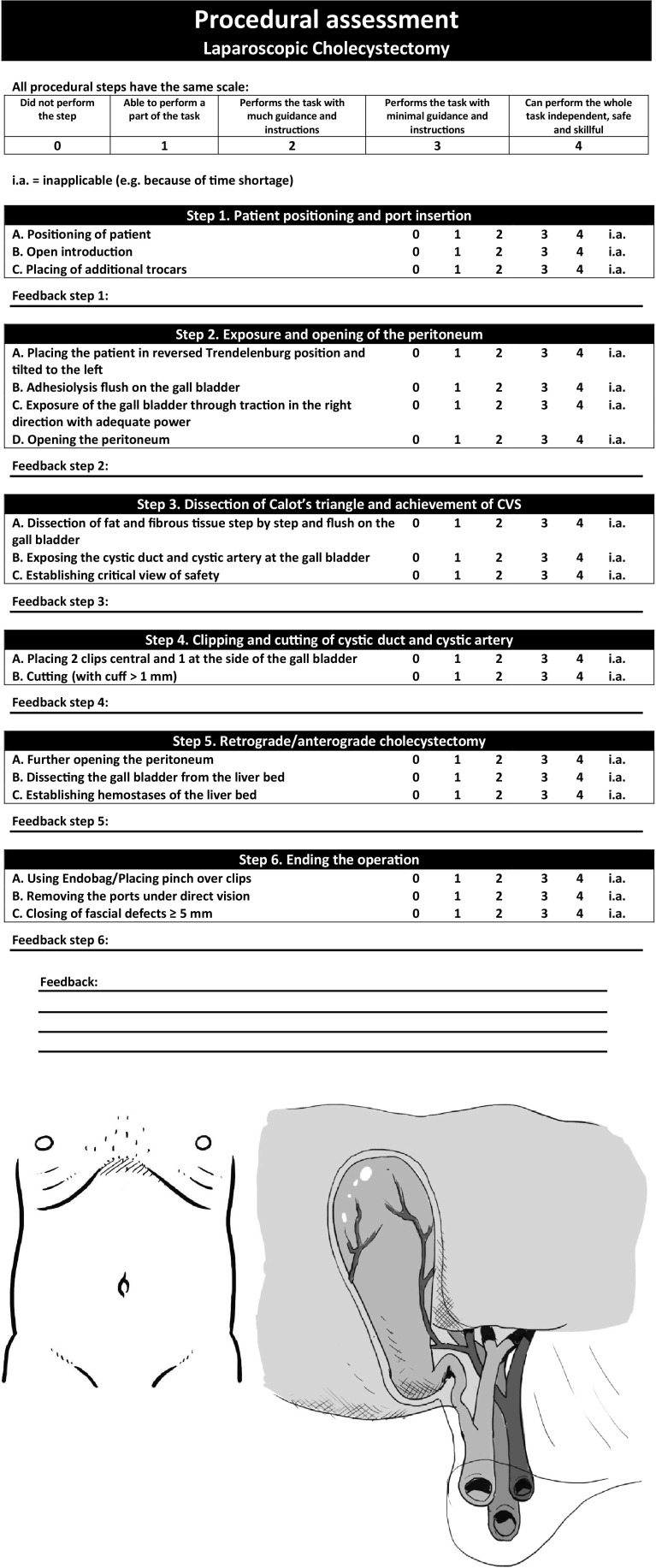
Independence-scaled procedural assessment form: Key steps of a procedure composed with the Delphi methodology combined to a scale based on the amount of assistants a trainee needs

The independence-scaled procedural assessment for the LC was used in a pilot experiment in the OR and iteratively adjusted on the basis of feedback from trainees and supervising surgeons. The final version of the independence-scaled procedural assessment is displayed in Fig. 1.

### Subjects

To evaluate the validity and reliability of the GRSs and independence-scaled procedural assessment, blinded videos were made and assessed by raters. Videos were made until videos from subjects of three different skill levels were obtained: (1) a novice trainee with prior simulator training, but little experience in the OR (novice: *N* = 1–6), (2) an advanced beginner that understands the basic principles, but still has much to learn (intermediate: *N* = 7–15) and (3) a trainee that is almost at the point of being qualified to independently perform a procedure, but still operates under direct supervision (subcompetent: *N* > 15).

### Video recording and blinding

Video and audio recordings were made in the OR with the laparoscope. The communication between the trainee and the supervising surgeon was recorded with two tiepin microphones attached beneath their surgical gown. The recorded audio was used to subtitle the video and to identify the parts in which the supervising surgeon physically assisted or took over a part of the procedure with one or two hands. Verbal communication of the trainee to the supervisor was marked at the beginning of the written sentence with the abbreviation ‘trainee’ and of the supervisor to the trainee with the abbreviation ‘SV.’ Parts performed by the supervisor were made visible in the output video by displaying the abbreviation ‘SV right/left’ when the supervisor assisted the procedure with one hand and ‘SV’ when the supervisor took over with both hands. After subtitling the communication, the videos were muted to prevent voice identification of the trainee and surgeon.

### Materials

The communication was recorded with a Shure PG188 PG185 wireless tiepin microphone (Shure, Culemborg, Gelderland, The Netherlands) attached to the trainee and the supervising surgeon beneath their surgical gown. A M-audio M-track USB audio interface (M-audio, Cumberland, RI, USA) was used in combination with Audacity 2.0.5 software (Free Software Foundation Inc., Boston, USA) to record the transmitted audio on a laptop. Microsoft Windows Moviemaker version 6.0.6000.16386 (Microsoft Corporation, Redmond, USA) was used to synchronize the audio material to the video material, convert the communication to subtitles and mute the video. The final output videos were windows media files of 768 × 576 pixels, 1000 kb/s, 4:3 screen ratio and 25 frames/s. The video material was distributed among raters with USB sticks in envelopes together with the paper assessment forms randomized in order.

### Raters

Ten consultant surgeons and three senior surgical trainees (HSTs) from four different surgical departments from the North-East Netherlands were invited to participate in the video assessment. In the invitations, they were informed that the assessment would take approximately 2.5 h. The trainees were all in their 4–6th year. In the Netherlands, these are the postgraduate training years in which trainees are expected to be able to independently treat uncomplicated gallbladder disease, supervise trainees from the 1–3rd year in treating uncomplicated gallbladder disease and perform OSATS assessments of the trainees they have supervised.

Scrub nurses are highly experienced with surgical instruments, but are also familiar with technical requirements of surgeons in the OR. They have seen the total scope of surgical skill levels among trainees, and in the majority of cases, they possess more OR experience than the operating trainee. Therefore, next to the surgical participants, also six scrub nurses with working experience in MIS suites were invited to participate in the video assessment.

### Assessment instructions, calibration and incentives

In our earlier research with GOALS assessment, we found a relatively low reliability compared to other studies [11]. We hypothesized that the lack of exposure and/or training to the assessment method might be one of the contributing factors, as was seen in a series of other studies [6, 12, 13]. In this study, the video assessments were therefore preceded by an introduction in order to calibrate the raters in the following way: (1) The items on the assessment forms were explained, (2) raters were encouraged to use the full scales as much as possible, (3) raters were instructed to use their own opinion when rating with the independence-scaled procedural assessment, and (4) we attempted to calibrate the raters by giving a clear definition of the low and high end of the scale of the GRSs items with a 2-min operative videos of a novice (*N* = 1) and of a consultant surgeon (*N* > 100). We also have hypothesized in the same study that a lack of motivation to complete a comprehensive assessment lengthy operative video material might lead to unreliable measurements [11]. Therefore, those who completed the assessments were rewarded with a box of wine of around 85$.

### Support for implementation

To evaluate the support for implementation of the OSATS, GOALS and independence-scaled procedural assessment among the surgeons and HSTs six questions were proposed (Table 1). Five questions could be answered with a score between 1 and 5, with 1 = strongly disagree and 5 = strongly agree. In the 6th question, raters were asked whether they rated the assessment tool as a subjective or objective assessment method with 1 = subjective and 5 = objective.

**Table 1:** Questionnaire about OSATS, GOALS and independence-scaled procedural assessment

	Strongly disagree	Disagree	Neutral	Agree	Strongly agree
Gives a correct judgment about the competence to perform a specific procedure	1	2	3	4	5
Leads to an unnecessary administrative burden	1	2	3	4	5
Should be used in clinical practice	1	2	3	4	5
Helps in the acquirement of procedural knowledge and skills	1	2	3	4	5
Should also be made for other laparoscopic procedures	1	2	3	4	5
Is objective or subjective	Subjective	Between neutral and subjective	Neutral	Between neutral and objective	Objective

### Statistical analysis

To be able to compare the different assessment methods and to correct for the missing items in GRS ratings and missing and inapplicable items in the independence-scaled procedural assessment score ratings, the ratings were calculated into a standardized percentage score with the formulas:

1. Procedural assessment score = [total score/(max. score − 4 × *N*_inapplicable_ − 4 × *N*_missing_)] × 100
2. GRS score = [(total score − (min. score − *N*_missing_)) /(max. score − (min. score − *N*_missing_) − 5 × *N*_missing_)] × 100

In the independence-scaled procedural assessment, the items ‘positioning of patient,’ ‘open introduction’ and ‘closing of wounds’ were not assessed because they were not captured on the video images of the laparoscopic camera.

Validity of the assessment tools was estimated by evaluating whether the increase in experience level between trainees in the videos led to a significant increase in performance score with the Friedman’s two-way analysis of variance by ranks. If a significant difference was observed between the video scores, the scores of video 1 and 2 and the scores of video 2 and 3 were compared with the Wilcoxon signed-rank test.

The reliability of an assessment tool is dependent on the amount of agreement between ratings of different raters and of crucial importance in high-stakes examinations. The reliability was calculated with the ICC. For a detailed discussion of different models to calculate the ICC, we refer to the publications of Shrout & Fleiss, McGraw & Wong and Hallgren [14–16]. In this study, the absolute agreement two-way random-effects model for single measures (AA-ICC 2,1) and the consistency agreement two-way mixed-effects model for single measures (CA-ICC 3,1) of the ICC were chosen. The values that are used to classify the ICC are random in nature and should be adapted to the purpose of the measurement instrument. To evaluate the assessment methods for the purpose of summative assessment, a cutoff value of 0.8 was used for the total score of the assessment method [4, 17]. For interpretation of the reliability of the individual items, the following cutoff values were used: ‘moderate’ (0.21–0.40), ‘reasonable’ (0.41–0.60), ‘good’ (0.61–0.80) and ‘almost perfect’ (0.81–1.00).

In the evaluation of feasibility, the assessment methods were compared with the Friedman test. If a statistically significant difference was observed, the assessment methods were mutually compared with the Wilcoxon signed-rank test.

All statistical analyses were performed with SPSS 20.0.0.1 (SPSS, Chicago, IL, USA). In all analyses, a *p* value of <0.05 (two-sided) was considered statistically significant. The Holm–Bonferroni method was applied to correct *α* for familywise error in the case of multiple testing.

## Results

### Videos

Three videos that met the assessment requirements were synchronized, subtitled and blinded. The number of LCs performed, year of training and OSATS score of trainees of the videos are given in Table 2. No significant difference in level of difficulty was observed between the three videos (*p* = 0.879, Friedman test).

**Table 2:** Characteristics of the three videos used for the blinded video assessment to estimate the reliability of the OSATS, GOALS and procedural assessment

	Caseload of trainee	Average percentage of OSATS score (%)	Training year	Time	Difficulty	Supervising surgeon
Novice	3	35	1	1:23	2 [1–3]	A
Intermediate	9	62	2	0:51	2 [1–3]	B
Subcompetent	27	88	3	0:43	2 [1–3]	B

The OSATS score is the mean of the live observation OSATS score achieved on the previous LC, the LC that was used for the video and the subsequent LC. The difficulty score is the median score and range on item 6 ‘Level of difficulty’ of the GOALS video assessments

### Raters

The surgeons and HSTs (group A) had performed a minimum of 50 LCs, and the scrub nurses (group B) had assisted a minimum of 50 LCs. Three surgeons were excluded in group A: Two surgeons could not participate in the assessment because of time shortage, and one rater was excluded because 4 of the 9 assessment forms were filled in with identical scores on all items, indicating an incomprehensive assessment. In the residual ratings, the maximum number of assessment forms with identical scores on all items was two.

### Validity

**Fig. 2:**
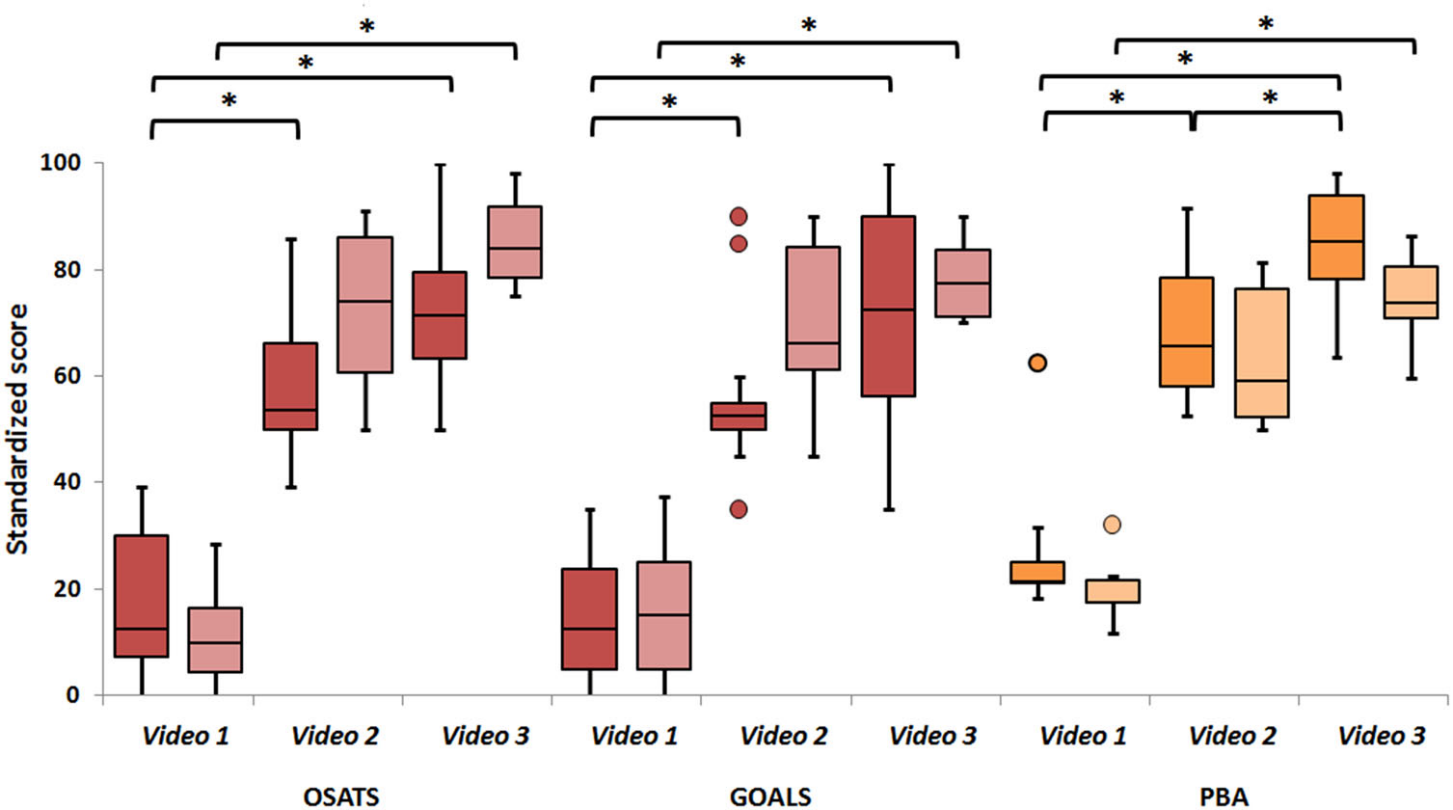
Validity of the independence-scaled procedural assessment and GRSs. Procedural assessment and GRSs scores improved significantly with surgical experience (OSATS *p* = 0.001, GOALS *p* < 0.001, Procedural assessment *p* < 0.001). However, the independence-scaled procedural assessment was the only one of the three assessment methods that could differentiate between the video of the intermediate and sub competent trainee among the surgical raters (*p* = 0.005)

Boxplots of the scores of group A and B are shown in Fig. 2. In group A, the median OSATS score was 12.5 [0.0–39.3] for video 1, 53.6 [39.3–85.7] for video 2 and 71.4 [50.0–100.0] for video 3 (*p* = 0.001). A significant difference was observed between video 1 and 2 (*p* = 0.005), but not between video 2 and 3 (*p* = 0.083). The median GOALS score was 12.5 [0.0–35.0] for video 1, 53.8 [35.0–90.0] for video 2 and 72.5 [35.0–100.0] for video 3 (*p* < 0.001). A significant difference was observed between video 1 and 2 (*p* = 0.005), but not between video 2 and 3 (*p* = 0.096). The median procedural assessment score was 22.4 [18.3–62.5] for video 1, 65.6 [52.5–91.7] for video 2 and 85.4 [63.5–98.2] for video 3 (*p* < 0.001). In contrast to the GRSs, a significant difference was observed between video 1 and 2 (*p* = 0.005) and between video 2 and 3 (*p* = 0.005).

In group B, the median OSATS score was 9.8 [0.0–28.6] for video 1, 74.1 [50.0–91.1] for video 2 and 83.9 [75.0–98.2] for video 3 (*p* = 0.006). No significant difference was observed between video 1 and 2 (*p* = 0.028) and video 2 and 3 (*p* = 0.115). The median GOALS score was 15.0 [0.0–37.5] for video 1, 66.3 [45.0–90.0] for video 2 and 77.5 [70.0–90.0] for video 3 (*p* = 0.009). No significant difference was observed between video 1 and 2 (*p* = 0.027) and between video 2 and 3 (*p* = 0.293). The median procedural assessment score was 21.7 [11.7–32.1] for video 1, 59.2 [50.0–81.3] for video 2 and 73.8 [59.6–86.5] for video 3 (*p* = 0.009). No significant difference was observed between video 1 and 2 (*p* = 0.028) and between video 2 and 3 (*p* = 0.173).

The median scores of the OSATS, GOALS and independence-scaled procedural assessment items of group A are given in Tables 3, 4 and 5. In independence-scaled procedural assessment scores, the scores for video 2 in step 4 ‘clipping and transection of the cysticus and artery’ were excluded, because the cystic duct was too large to be clipped with a clip of normal size. A significant difference between video 1 and 2 and video 2 and 3 was only observed in OSATS item 2 ‘time and motion.’

**Table 3:** Standardized score and range of OSATS items for video 1–3 of group A

OSATS	Video 1	Video 2	Video 3	*p*(1–2–3)	*p*(1–2)	*p*(2–3)
1. Respect for tissue	2.0 [1.0–4.0]	3.0 [2.0–5.0]	4.0 [2.0–5.0]	0.002*	0.007*	0.666
2. Time and motion	1.5 [1.0–3.0]	3.0 [2.0–4.0]	3.5 [3.0–5.0]	<0.001*	0.007*	0.025*
3. Instrument handling	1.0 [1.0–3.0]	3.0 [3.0–5.0]	4.0 [3.0–5.0]	<0.001*	0.004*	0.305
4. Knowledge of instruments	2.0 [2.0–3.0]	3.5 [3.0–5.0]	4.5 [3.0–5.0]	0.001*	0.011*	0.084
5. Use of assistants	1.0 [1.0–4.0]	3.0 [2.0–4.0]	3.5 [2.0–5.0]	<0.001*	0.006*	0.035
6. Flow of operation	1.0 [1.0–2.0]	3.0 [1.0–4.0]	4.0 [2.0–5.0]	0.001*	0.008*	0.058
7. Knowledge of procedure	2.0 [1.0–3.5]	4.0 [3.0–5.0]	4.0 [3.0–5.0]	<0.001*	0.005*	0.194

*p* values were calculated with the Friedman test, and differences between video 1 and 2 and video 2 and 3 were evaluated with the Wilcoxon test. The Holm–Bonferroni method was applied to correct the significance level

* Statistical significant

**Table 4:** Standardized score and range of GOALS items for video 1–3 of group A

GOALS	Video 1	Video 2	Video 3	*p*(1–2–3)	*p*(1–2)	*p*(2–3)
1. Depth perception	1.0 [1.0–4.0]	3.0 [2.0–5.0]	4.0 [2.0–5.0]	0.005*	0.007*	0.589
2. Bimanual dexterity	2.0 [1.0–2.0]	3.5 [2.0–5.0]	4.0 [3.0–5.0]	<0.001*	0.007*	0.058
3. Efficiency	1.0 [1.0–2.0]	3.0 [3.0–4.0]	4.0 [3.0–5.0]	<0.001*	0.004*	0.096
4. Tissue handling	2.0 [1.0–3.0]	3.0 [2.0–5.0]	4.0 [2.0–5.0]	0.005*	0.017*	0.341
5. Autonomy	1.0 [1.0–2.0]	2.5 [1.0–4.0]	4.0 [1.0–5.0]	0.001*	0.007*	0.047

*p* values were calculated with the Friedman test, and differences between video 1 and 2 and video 2 and 3 were evaluated with the Wilcoxon test. The Holm–Bonferroni method was applied to correct the significance level

* Statistical significant

**Table 5:** Standardized score and range of procedural assessment items for video 1–3 of group A

Independence-scaled procedural assessment	Video 1	Video 2	Video 3	*p*(1–2–3)	*p*(1–2)	*p*(2–3)
1. Positioning and introduction of the trocars	25.0 [0.0–75.0]	75.0 [50.0–100.0]	87.5 [75.0–100.0]	<0.001*	0.007*	0.096
2. Exposition gallbladder and opening of peritoneum	33.3 [18.8–43.8]	75.0 [41.7–100.0]	91.7 [66.7–100.0]	<0.001*	0.005*	0.042
3. Dissection of Calot’s triangle	12.5 [0.0–66.7]	43.8 [25.0–75.0]	66.7 [25.0–91.7]	<0.001*	0.005*	0.192
4. Clipping and transection of the cysticus and artery	12.5 [12.5–75.0]		100.0 [75.0–100.0]	–	0.004*	
5. Retrograde/anterograde cholecystectomy	29.2 [16.7–75.0]	75.0 [33.3–100.0]	100.0 [75.0–100.0]	<0.001*	0.011*	0.026
6. Extraction of gallbladder and closing of wounds	25.0 [0.0–50.0]	75.0 [75.0–100.0]	93.8 [75.0–100.0]	<0.001*	0.005*	0.482

*p* values were calculated with the Friedman test, and differences between video 1 and 2 and video 2 and 3 were evaluated with the Wilcoxon test. The Holm–Bonferroni method was applied to correct the significance level

* Statistical significant

### Reliability

The reliability of the AA-ICC and CA-ICC of the OSATS, GOALS and independence-scaled procedural assessment scores and their individual items are given in Tables 3, 4, 5, 6, 7 and 8. The AA-ICC of the total OSATS score was 0.78 in group A and 0.91 in group B (Table 6). Most OSATS items had a good or almost perfect reliability in both groups, except for the items respect for tissue and use of assistance. Interestingly, the two items ‘use of assistance’ and ‘instrument handling’ attained an AA-ICC and CA-ICC of ≥0.90 in group B.

The AA-ICC of the total GOALS score was 0.74 in group A and 0.85 in group B. The AA-ICC and CA-ICC of the items ‘depth perception’ and ‘tissue handling’ were reasonable in group A (Table 7).

**Table 6:** AA-ICC and CA-ICC of standardized total OSATS score and the standardized score of the items of the OSATS

Item	Group A + B		Group A		Group B	
	AA-ICC	CA-ICC	AA-ICC	CA-ICC	AA-ICC	CA-ICC
1. Respect for tissue	0.50	0.51	0.47	0.49	0.49	0.46
2. Time and motion	0.71	0.76	0.71	0.77	0.74	0.75
3. Instrument handling	0.78	0.80	0.71	0.70	0.90	0.94
4. Knowledge of instruments	0.76	0.79	0.72	0.72	0.82	0.90
5. Use of assistants	0.70	0.80	0.58	0.74	0.90	0.92
6. Flow of operation	0.74	0.77	0.64	0.68	0.88	0.89
7. Knowledge of procedure	0.76	0.73	0.66	0.65	0.86	0.83
Total	0.83	0.84	0.78	0.79	0.91	0.92

All ICCs were statistically significant (*p* < 0.05)

**Table 7:** AA-ICC and CA-ICC of standardized total GOALS score and the standardized score of the items of the GOALS

Item	Group A + B		Group A		Group B	
	AA-ICC	CA-ICC	AA-ICC	CA-ICC	AA-ICC	CA-ICC
1. Depth perception	0.64	0.71	0.49	0.53	0.84	0.95
2. Bimanual dexterity	0.78	0.83	0.76	0.78	0.83	0.90
3. Efficiency	0.86	0.89	0.87	0.87	0.84	0.91
4. Tissue handling	0.56	0.56	0.49	0.46	0.59	0.64
5. Autonomy	0.66	0.69	0.60	0.67	0.78	0.72
6. Level of difficulty	NS	NS	NS	NS	NS	NS
Total	0.79	0.81	0.74	0.75	0.85	0.89

All ICCs were statistically significant (*p* < 0.05)

**Table 8:** AA-ICC 2,1 and CA-ICC 3,1 of standardized total procedural assessment score and the standardized score of the items of the procedural assessment

Procedural step	Group A + B		Group A		Group B	
	AA-ICC	CA-ICC	AA-ICC	CA-ICC	AA-ICC	CA-ICC
1. Positioning and introduction of the trocars	0.82	0.80	0.79	0.77	0.89	0.86
2. Exposition gallbladder and opening of peritoneum	0.79	0.76	0.82	0.80	0.71	0.66
3. Dissection of Calot’s triangle	0.45	0.59	0.50	0.63	0.52	0.52
4. Clipping and transection of the cysticus and artery	0.92	0.94	0.90	0.92	0.97	0.97
5. Retrograde/anterograde cholecystectomy	0.74	0.75	0.75	0.74	0.67	0.71
6. Extraction of gallbladder and closing of wounds	0.89	0.88	0.86	0.84	0.92	0.92
Total	0.85	0.87	0.84	0.86	0.87	0.86

In step 1, ‘positioning’ (=preoperative positioning) was not assessed, and in step 6, ‘closing of wounds’ was not assessed. All ICCs were statistically significant (*p* < 0.05)

The AA-ICC of the total independence-scaled procedural assessment score was 0.84 in group A and 0.87 in group B. The procedural step dissection of Calot’s triangle had a reasonable ICC, and only the CC-ICC in group A was good (Table 8).

When group B was added to group A, the ICCs of the total scores and items were higher than that of group A in all three assessment methods, except for dissection of Calot’s triangle (Table 8).

### Support for implementation

**Fig. 3:**
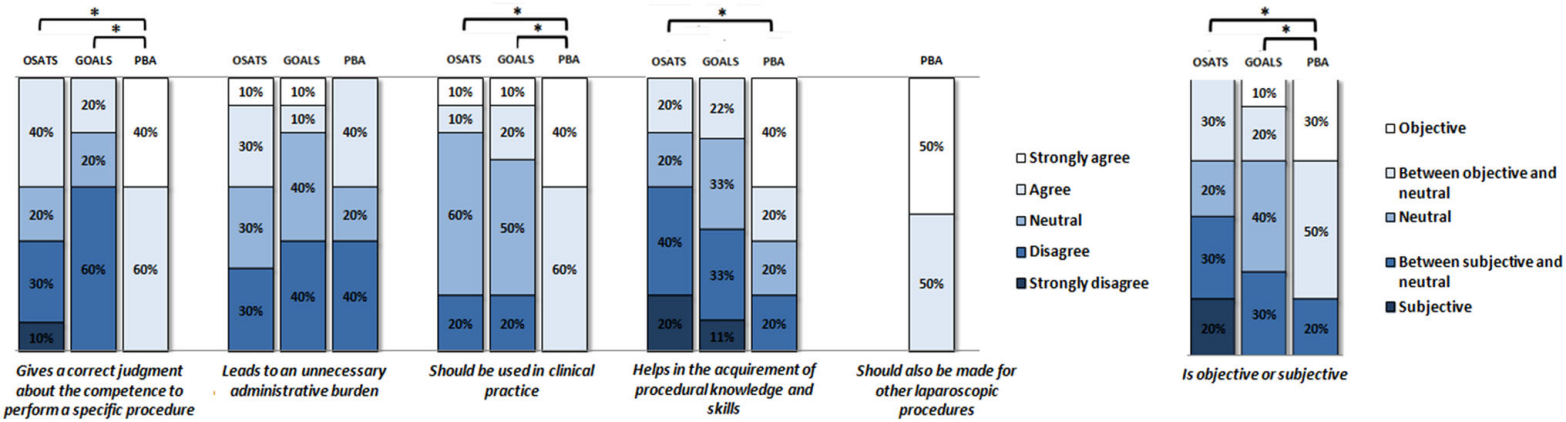
Results of the questionnaire distributed among surgeons and higher surgical trainees

Seven surgeons and three surgical trainees completed the questionnaire (Fig. 3). All shared the opinion that the independence-scaled procedural assessment score gives a correct judgment of competency in a specific procedure, compared to six for the OSATS and four for the GOALS (*p* = 0.001). A significant difference was observed between the independence-scaled procedural assessment and the GRSs (*p* = 0.011 for OSATS, *p* = 0.005 for GOALS). Four raters found the independence-scaled procedural assessment an unnecessary administrative burden, compared to four for the OSATS and two for the GOALS (*p* = 0.692). They all thought that the independence-scaled procedural assessment should be used in clinical practice, compared to two for the OSATS and three for the GOALS (*p* = 0.005). A significant difference was observed between the independence-scaled procedural assessment and the GRSs (*p* = 0.018 for OSATS, *p* = 0.010 for GOALS). Six raters agreed on the statement that the independence-scaled procedural assessment could help in the acquirement of procedural knowledge and skills compared to two for the OSATS and two (two out of nine because of missing data from one rater) for the GOALS (*p* = 0.025). A significant difference was only observed between the independence-scaled procedural assessment and the OSATS in this question (*p* = 0.009). Eight observers considered the independence-scaled procedural assessment to be objective compared to three for the OSATS and three for the GOALS (*p* = 0.007). A significant difference was observed between the independence-scaled procedural assessment and the GRSs (*p* = 0.015 for OSATS, *p* = 0.023 for GOALS). All participants encouraged a reproduction of the independence-scaled procedural assessment for other laparoscopic procedures.

## Discussion

Although GRSs have proven its value in formative feedback in training, controversy exists about their usefulness in procedure-specific assessment and certification for independent surgical treatment of uncomplicated disease. A multicenter blinded study was conducted to estimate the validity, reliability and feasibility of the procedural assessment and two GRS of which one, the OSATS, is an integral part of surgical training in the Netherlands. A procedural assessment for the LC was created by linking the previously published operative key steps to an independence scale to create a procedural assessment [9]. Three blinded and subtitled videos of trainees of different skill levels were assessed with the independence-scaled procedural assessment, OSATS and GOALS by surgeons, senior surgical trainees and scrub nurses. In addition, a questionnaire was completed that aimed to measure the support for implementation of the independence-scaled procedural assessment, OSATS and GOALS in practice.

### Validity

The independence-scaled procedural assessment, OSATS and GOALS all showed a significant improvement in assessment scores with increasing experience levels. This supports the results of previous studies that have evaluated the validity of GRSs and independence-based procedural assessment [3, 6, 10, 11]. However, in this study, the independence-scaled procedural assessment was the only one of the three assessment methods that could differentiate between the video of the intermediate and subcompetent trainee among the surgical raters. This indicates that the independence-based procedural assessment is the most sensitive assessment method to measure skill level in the performance of a procedure and is in line with recent studies that studied independence scales. For instance, Glarner et al. [10] used an independence scale as an indirect measure of the skill level of the surgeon for assessment of a hemicolectomy. Their independence-scaled procedural assessment showed an increase in performance level in residents during a colorectal rotation, while the GRSs showed little to no increase during the rotation. Furthermore, Cornelis et al. have shown that the so-called Alphabetic Summary Scale, an independence-based rating scale, had a higher discriminating power than a modified form of the OSATS and an overall performance scale for assessment of osteosynthesis of proximal femoral fractures [3].

Next to the higher sensitivity, the independence-scaled procedural assessment also has the advantage of providing educators and trainees with the opportunity to preoperatively discuss which procedural steps will be performed by the trainee and assessed by the supervisor. This enables a stepwise expansion of the amount of steps performed by a trainee. GRSs lack the benefits of enabling stepwise teaching and the use of solely a GRS to assess operative competence and therefore probably do not optimally facilitate the teaching of procedural skills. The GRSs also lack an option for narrative (descriptive) feedback. We decided to include multiple options for giving narrative feedback in the independence-scaled procedural assessment, which makes it more suitable for giving feedback that is task specific and focused on the learning goals of a trainee [17].

### Reliability

This is the first blinded multicenter study that simultaneously investigates the reliability of GRSs and independence-based procedural assessment for a standard laparoscopic procedure. The patterns observed in the reliability analysis give valuable insights in the factors that influence reliability in the assessment of surgical competence.

Among the raters with surgical training, the reliability of the GRSs did not reach the threshold of 0.8. This finding is in line with the majority of studies that addressed the reliability of GRSs [4]. There are a series of factors that could have led to an inter-rater reliability below the threshold value. In the past, authors have argued that training might be of key importance in attaining reliable scores with GRSs [6, 11, 12]. Because the OSATS is an integral part of surgical training in the Netherlands, all surgical raters were familiar with this assessment method. However, some of the raters had never used the other two assessment methods to assess operative competence. We attempted to introduce raters to the key elements of the assessment methods and to calibrate them with short introductory videos prior to assessment. In both GRSs, the introduction and calibration did not lead to an acceptable reliability for summative assessment.

Assuming the introduction to assessment was done appropriately, the most likely remaining cause of not attaining the threshold is characteristics of the GRSs itself. The format of the GRSs, in particular the Likert scale, has been subject of discussion. Some authors even state that attaining a reliability of 0.80 is almost impossible when using a Likert scale [18]. The descriptions of the anchors show a possible weakness of the GRSs. The anchors contain words such as ‘frequently,’ ‘unnecessary’ and ‘inappropriate’ that are strongly susceptible to differences in interpretation, and the absence of descriptions on anchors with score two and four might increase subjectivity even more. The terminology and characteristics of the scale probably contribute to a barrier for attaining a high inter-rater reliability with GRSs.

In contrast to the GRSs, the independence-scaled procedural assessment showed an inter-rater reliability higher than 0.8 among surgeons, indicating that an independence-based procedural assessment tool is a suitable candidate for certification and authorization in the treatment of uncomplicated disease. This is in line with the observation of an ICC higher than 0.8 by Miskovic et al. [19] who evaluated independence-scaled procedural assessment in colorectal surgery and determined inter-rater reliability by correlating peer with self-assessments. It seems that assessment of a series of procedural key steps, on which consensus has been achieved, compels raters to look at specific elements of operative competence and thereby gives less room for subjectivity. The high inter-rater reliability could theoretically also have been caused by a higher between-subjects variance in the independence-scaled procedural assessment: If the performance level of trainees with different experience levels measured with a procedural assessment shows more variance than when assessed with a global assessment method, the reliability of the former would automatically tend to increase based on the calculation model of the ICC [20]. However, comparison of the between-subjects mean square of the independence-scaled procedural assessment and GRSs did not indicate that this was the case.

Although the total independence-scaled procedural assessment scores showed a high reliability, subjectivity was not totally expelled. This was especially evident in the inter-rater reliability of the dissection of Calot’s triangle. Interestingly, among surgeons the CA-ICC was good, indicating that part of the error variance is caused by some clinical supervisors being more stringent than others in the assessment of this step. To increase the inter-rater reliability in this procedural step, a more detailed procedure characterization with the inclusion of procedure errors could have been included as has been done by others [21, 22]. However, several researchers in the domain of performance appraisal have proposed an alternative view on inter-rater reliability that might be relevant in the assessment of the dissection of Calot’s triangle. This view has been described by Govaerts et al. [23] as the ‘constructivist social-psychological approach.’ One of the central themes of this perspective is that ‘raters from different perspectives may rate differently because they observe different aspects of performance, and differences in ratings may very well reflect true differences in performance.’ The dissection of Calot’s triangle is the most complex and therefore the most technically demanding step. Because the high difficulty requires a mixture of technical behaviors in the trainee, the rater has to make a decision on which aspect of technical behavior of the trainee to rate during the observation of the behavior during this step and also has to decide on which way it will be assessed. These decision processes are influenced by knowledge, operative experiences and the content and characteristics of the interactions with supervisors who supervised the rater (socialization). Thus, although the ratings do not agree in the assessment of the dissection of Calot’s triangle, they might all be equally valid, because they are funded on the individual professional experience and understanding of the raters. If so, this could have the implication that a summative assessment of a trainee would not be based on the assessment of one rater, but on multiple raters, not to achieve a more reliable numerical score, but to achieve a more complete picture of the level of surgical skills [23]. For instance, a trainee would only be considered eligible for certification in the independent treatment of uncomplicated gallbladder disease if a specific cutoff score is achieved on two laparoscopic cholecystectomies, each supervised by independent consultant surgeon that did not have prior communication about the training performance of the trainee.

At last, when the ratings of the scrub nurses were combined with those of the surgically trained raters, almost all the reliability coefficients of the total scores and item scores increased slightly, indicating that, in line with the study of Beard et al. [24], there is agreement between the assessment of scrub nurses and surgeons. Although the authorization of surgical trainees in the independent treatment of patients with uncomplicated disease should be reserved for clinical supervisors, these findings indicate that scrub nurses can be of contributive value in the assessment of operative competence of trainees.

### Support for implementation

In the questionnaire, there was strong support for implementation of the independence-scaled procedural assessment into practice. Although we did not give an extensive description on what can go good and what can go wrong, it was considered to give a more correct judgment of procedural skills than the GRSs. Participants were also asked to rate the assessment methods on objectivity. The median score of objectivity for the OSATS and for the GOALS in this study was 2.5 and 3.0, respectively, which is similar to the median score of 3.0 observed by Hiemstra et al. [5] on the same question for the OSATS among gynecologists and gynecological residents. However, eight out of ten considered the independence-scaled procedural assessment to be objective (median score = 4.0). Furthermore, all participants encouraged reproduction of the independence-scaled procedural assessment for other laparoscopic procedures. These findings are in line with the findings of Beard et al. [8] who have shown a higher acceptability and satisfaction of their procedure-based assessment than for the OSATS among trainees and clinical supervisors.

### Development of procedural assessment

**Fig. 4:**
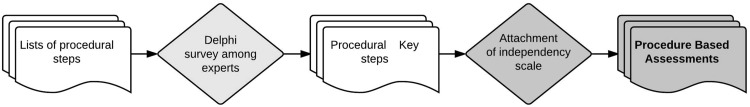
Two-step system for the development of procedure-specific assessments

Although more studies by other research institutions are necessary to confirm the results, on the basis of the results, a two-step system seems to be a viable option for the development for procedural assessments (Fig. 4). The first step consists of using a regional expert panel to reach consensus on the key steps of a procedure. The procedural steps that are considered of key importance in a procedure can vary regionally and internationally. By using the opinion of experienced surgeons involved in surgical training programs within the region, the procedural steps will be relevant and important to those using it (content validity). In the second step, an independence scale is attached to the key steps to assess operative competence.

An alternative to the second step would be to give elaborate descriptive terms of how the key steps of a procedure should be performed or to insert some form of error analysis in the assessment as has been done by others [21, 22, 25–30]. However, error-based assessment might be limited in assessment above the performance level of what Wentink et al. [31] call skill and rule-based behavior. The higher levels of cognition, by Wentink et al. [31] described as ‘knowledge-based behavior,’ are used for the development and execution of strategies to deal with unfamiliar situations during surgery. This level of behavior moves more to the foreground in the last part of the learning curve, the phase in which skill- and rule-based behavior has been largely acquired, but reasoning might need some important adjustments at times. The independence-scaled assessment method gives supervisors the freedom of assessing the level of knowledge-based behavior on the basis of their professional judgment of unfamiliar situations and the adequacy of the trainee’s response on these situations. This aspect of assessment is essential in identifying trainees who are ready for independent surgical treatment of patients. Future studies that compare independence-based procedural assessment, error-based procedural assessment and checklist-based procedural assessment in terms of validity, reliability and feasibility could provide more insight on the strengths and weaknesses of each of these assessment methodologies.

### Limitations

There are some limitations to our study that have to be addressed. First, the videos were blinded but not randomized. Not using a random sequence could have introduced bias in the assessment. However, as some raters rated video 3 lower than video 2, we do not think that not randomizing the videos affected the raters significantly.

Second, the error variance could have been lower in the independence-scaled procedural assessment because the raters simply did not use their own opinion but adopted that of the supervising surgeon of the video, resulting in a higher reliability than the GRSs. The scrub nurses might be particularly susceptible to this, but the reliability of the independence-scaled procedural assessment of the scrub nurses was similar to that of the GRSs. Therefore, there is no indication that this phenomenon might have artificially increased the reliability of the independence-scaled procedural assessment.

Third, although the literature agrees about using 0.80 as a threshold when assessing reliability for high-stakes examinations, the use of a somewhat arbitrary number as a threshold is arguable. A threshold of 0.80 only means that 80 % of the difference between ratings is attributable to true variance and the remaining is caused by random error, rater error and/or other sources of error. Despite this weakness, the threshold is one of the few tools available to identify assessment methods with an inter-rater reliability satisfactory for summative assessment and is strongly adhered to in the surgical literature [4].

Fourth, no attempts were made to define cutoff values for the independent surgical treatment of uncomplicated gallbladder disease. Research is currently being conducted in our center to collect the required data to establish cutoff values for the identification of competent trainees.

Fifth, after the achievement of a certain skill level, a decay effect has been observed of the acquired skills [32–34]. The amount of decay that arises is dependent on two variables: (1) How familiar the trainee is with the skills and (2) The amount of time that has passed since the last performance. Although we expect that the independence-scaled procedural assessment is able to identify the level of procedural skills required for the LC, no statements can be made about the number of procedures that have to be performed in order to minimize the decay effect or the length of time the acquired level of procedural skills will be retained. Furthermore, it could be that the rather verbal passive form of training necessary for adequate independence-scaled procedural formative assessment, increases the retention of skills as described by the guidance hypothesis [35, 36].

Finally, assessment of non-technical skills such as medical knowledge, communication skills and clinical judgment was not included in this study. Non-technical skills are critical components of operative care and should complement assessment of technical skills when surgical competence is addressed.

## Conclusion

In conclusion, a valid and reliable procedural assessment method can be developed by linking the key steps of a procedure, composed with the Delphi methodology, to an independence-based scale. The validity and reliability of the independence-scaled procedural assessment exceeded that of the global rating scales in the blinded assessment of a laparoscopic cholecystectomy. Among the group of raters with surgical training, an inter-rater reliability above the threshold value of 0.80 was only observed in the procedural assessment. Moreover, the participants expressed strong support for the use of the independence-scaled procedural assessment in clinical practice and encouraged its reproduction for other procedures. This study demonstrates that independence-scaled procedural assessment can be a valuable assessment tool and appears to comply with the requirements of use for procedural certification.
